# “HTLV-I Infection” Twenty-Year Research in Neurology Department of Mashhad University of Medical Sciences

**Published:** 2013-03

**Authors:** Ali Shoeibi, Mohammdmahdi Etemadi, Amir Moghaddam Ahmadi, Mona Amini, Reza Boostani

**Affiliations:** 1 Assistant Professor of Neurology, Mashhad University of Medical Sciences; 2 Professor of Neurology, Mashhad University of Medical Sciences; 3 Neurologist, Rafsanjan University of Medical Sciences; 4 Resident of Neurology, Mashhad University of Medical Sciences

**Keywords:** HTLV-I, Iran, Spastic paraparesis, Tropical

## Abstract

Human T-cell lymphotropic virus (HTLV) types 1 and 2 belong to the Oncorna group of retroviridae, a large family of viruses, grouped initially by pathogenic features, but later revised on the basis of genome structure and nucleotide sequence. HTLV-I was the first discovered human retrovirus to be associated with a malignancy in 1980. The malignancy, first described by Uchiyama and co-workers in southwestern Japan, was named Adult T-cell Leukemia/Lymphoma (ATL) and characterized with cutaneous and respiratory involvement, hepatosplenomegaly, lymphadenopathy and various metabolic abnormalities such as hypercalcemia. The HTLV-I has been known to be endemic to certain parts of Iran like the province of Khorasan in the northeast since 1990, with a 2.3% prevalence rate of infection. The main manifestations of HTLV-I infection are neurologic and hematologic (such as ATL) disorders, but it has also other manifestations such as uveitis, arthritis, dermatitis, vitiligo and lymphocytic alveolitis. Its main neurologic manifestation is a chronic progressive myelopathy that is referred to HTLV-I Associated Myelopathy (HAM) in Japan and Tropical Spastic Paraparesis (TSP) in Caribbean. But other disorders such as peripheral neuropathy, polyradiculoneuropathy, myopathy, peripheral facial paresis, and so on have been reported too. In this review we wish to give some brief information on the different aspects (including epidemiology, pathogenesis and pathology, clinical findings, and treatment) of HTLV-I infection according to our twenty-year researches. The department of neurology of Mashhad University of Medical Sciences has been a pioneer in researches on HTLV-I in the last twenty years.

## Epidemiology

HTLV-I infection is endemic in tropical areas such as South America, Central Africa, Caribbean area, south of Japan, and the Middle East (The province of Khorasan, Iran, south of India, and West Africa). It has been estimated that 10 to 20 million people around the world are infected with HTLV-I (1-7). Its prevalence in Mashhad, the capital city of the province of Khorasan, has been estimated as 2-3% in the whole population (7-10), and 0.7% to 1.16% in blood donors. According to the study by Farid *, et al,* in 1992, the prevalence of HTLV-I infection in Khorasan Razavi was 2.3% (12). This was reported 1.45% among blood donors in 1994 in a study by Shirdel* et al*. In Shirdel’s study, the prevalence of virus infection was 2.6% among women and 1.3% among men (11). However, it seems that the prevalence rates are decreasing in recent years.

Levels of infection are high in endemic populations; however, most seropositive individuals are asymptomatic and less than 2% develop neurological disease, mostly HAM/TSP. It has been proposed that both viral and host factors are important in increased susceptibility for neurological disease. The most important factors are HTLV-I subgroups, proviral load, and HLA background (1, 7). Etemadi, Farid Hosseini *et al.* studied the prevalence of IL2 receptor in HTLV-I carriers, HAM/TSP patients, and control group in 1997 and showed that IL2 receptor prevalence was significantly higher in HAM/TSP patients and HTLV-I carriers than in control group. However there was no significant difference between HAM/TSP patients and HTLV-I carriers. This study also showed that there was not any meaningful difference among receptor prevalence with relation to age, sex, and disease severity and duration (11). Farid Hosseini, Nikbin *et al.* studied types of HLA in families of HTLV-I patients in Khorasan and found out that the most common HLA type was HLA-430 and the most common antigen was A26. In this study, 40% of first-degree relatives of HTLV-I patients were carriers of the virus, which was ten times more prevalent than the same population in that province. Levels of serum antibodies to HTLV-I are higher in healthy adults living in endemic regions compared with non-endemic regions, in ATL patients compared with healthy adults, and also in healthy relatives of ATL patients compared with other people in that region (7).

 Like other retroviruses, HTLV-I is transmitted by sexual, parenteral, or vertical (mostly through breast feeding, and very rarely in utero) transmission (12). In Iran, like many other countries worldwide, all blood supplies are screened for HTLV-I (13). It is important to note that the transmission of HTLV-I is highly cell-associated, meaning it requires the infected live cells to be transmitted, not the free virus particles; for example, the fresh frozen plasma of a seropositive individual does not lead to the transmission of the infection. It has been proposed by some researchers that most of the vertical transmissions occur via the T-lymphocytes filled with HTLV-I particles in breast milk (approximately 1000 cells per milliliter); thus, the risk of transmission will not be increased with breastfeeding if the live cells can be omitted via freezing and re-warming (12). In 2004, Etemadi, *et al,* showed that 14.75% of HAM/TSP patients had a history of blood transfusion (11). This rate was 15.3% in the study by Etemadi, *et al* 2005, 22% in the study by Etemadi *et al* in 2007, and 4.2 % in the study by Boostani *et al* in 2010 (11). 

 The risk of transmission via breastfeeding is approximately 16 to 30 percent. The rate of transmission depends on the duration of breastfeeding (14.4% for more than 7 months, versus 4.4-5.7% for less than 7 months), and maternal proviral load. Although transmission in utero is possible, it is very rare (1, 6). Goodarzi *et al* reported breast-feeding as the most important route of transmitting HTLV-Iinfection in Khorasan in 1993 (11). Farid Hosseini *et al* in 1997, and Boostani *et al* in 2010, showed that 100% of HAM/TSP had the history of breast-feeding for more than one year and this rate was 92.9% in the study of Etemadi *et al* in 2005 (11), Farid *et al* investigated HTLV-I infection in pregnant women and measured the rate of positivity of antiviral antibody in umbilical cord blood sample in 1994. The prevalence of HTLV-I infection in pregnant women in Mashhad was 3.3%. In this study, all the children of infected mothers had antivirus antibody in their umbilical cord blood sample. There was a meaningful correlation between HTLV-I infection and the number of parities, low socio-economic status, number of abortions, mother’s age at the time of parity, and abnormality of placenta. No significant correlation was found between HTLV-I infection and children’s birth weights, infancy disorders and APGAR score at one and five minutes after deliveries. Induced labor or prolonged delivery was more common in HTLV-I infected women than the general population, but considering low sample volume of the study, there was no statistical comparison (11). Etemadi *et al* in a study, in 1997, showed that 17.47% of HAM/TSP patients had a positive family history of HAM/TSP (11). In other studies by Etemadi *et al* in 2000, Boostani *et al * in 2003, Etemadi *et al* in 2005, Etemadi *et al* in 2007, Boostani *et al* in 2010, it was measured 9%, 19.67%, 15.1%, 24%, and 25% respectively (11).

 Transmission from male to female is significantly more common than transmission from female to male, with a risk of about 60.8% in contrast to 0.4%, respectively (1). The history of unsafe sexual contact was reported in 9.7% of HAM/TSP patients in a study in 2005 by Etemadi *et al* (11). None of the studies conducted in this centre reported any transmission of the virus via needle stick or haemodialysis.

## Pathology and pathogenesis

Regarding the pathogenesis of CNS involvement by HTLV-I, various mechanisms have been proposed. Direct attack of the virus to the neurons is not proved; however, the indirect contamination of nervous system by lymphocytes and autoimmune mechanisms (both humeral and cellular) is more suspected. Some cytokines such as interleukins, platelet activating factor (PAF), and tumor necrosis factor (TNF) have been found in serum of HAM/TSP patients. It seems that they have a role in demyelination in the nervous system. The relation of CD4-positive to CD8-positive T-cells is increased in these patients and the response of Natural Killer cells (NKs) is disrupted in ATL and HAM/TSP patients (7, 14-18).

 Osame has been suggested the immunological model that leads to HAM/TSP: 1- The activation of T-lymphocytes (especially CD4+) and macrophages, and expression of cytokines (such as TNF-α, IL-1β, and IFN-γ), 2- The infiltration of CD8+ cytotoxic T-cells in the nervous system, 3- The localization of HTLV-I in the nervous system, mainly inside the T-lymphocytes, 4- The recruitment of T-lymphocytes due to the increased leukocyte adhesion molecules (such as VCAM-4) on the endothelial surface of the vessels and also disruption of blood-brain-barrier with secretion of matrix-metalo-proteinases (MMPs), and 5- The axonal degeneration in the spinal cord (7). These immune responses seem to become activated as a result of interaction with retroviral env and tax proteins, having the greatest activity within the thoracic and less in lumbar spinal cord, which lead to predominant clinical findings in the lower limbs (1, 10).

 Pathological changes in HAM/TSP patients include a chronic inflammatory meningomyelopathy that is predominant in the thoracic spinal cord and leads to the degeneration of corticospinal, spinothalamic, and spinocerebellar pathways in the dorsal and lateral columns (16). Also, there are some cystic necrosis and atrophic changes in the spinal cord due to perivascular inflammatory reactions; some changes in anterior horn cells and arachnoid thickening occur, and demyelination involves dorsal column, pyramidal tracts, and posterior and anterior roots (16, 19). In 2010, Boostani, *,et al,* investigated somatosensory evoked potentials (SSEP) changes in median nerve in HAM/TSP patients and found a meaningful correlation between disease severity and N20 latency. This study showed a significant involvement of posterior spinal column and medial lemniscuses tracts of brainstem and thalamocortical tracts even if there were no signs of upper limb involvement in HAM/TSP patients (11).

*Clinical features*

Various neurological disturbances can be caused by HTLV-I infection, such as myelopathy, polyneuropathy, cranial neuropathy, optic neuritis, cerebellar ataxia, vertigo, nystagmus, dementia, myositis, and an ALS-like syndrome (1, 7, 16). 

*Myelopathy*

With an incubation period of 6 months to 20 years, the most common neurological disorder, HAM/TSP, is usually recognized in the third to fifth decade of life as a chronic progressive spastic paraparesis or myeloneuropathy. Rarely, acute cases, resembling transverse myelitis may occur (20, 21). It is more common in females, with a 2:1 to 3:1 ratio. The mean age of disease presentation in studies of Etemadi,* et al,* 2000, 2003, 2005, 2007, was 40, 46.9, 45.9 and 41.9 years old respectively (11). This was measured 45.6 years old in a study by Boostani, *et al * (11). In all studies, female to male proportion in HAM/TSP patients was 3 to 1 (12). However, this proportion was 2 to 1 in a study conducted by Etemadi *et al *in 1997 (11). The interval between developing the symptoms and diagnosis of the disease ranged between 1-10 years in male patients and 1-20 years in female patients in a study by Etemadi *et al* (11) in 1994. The mean period between symptoms onset and disease diagnosis was reported 9.92 years by Farid Hosseini *et al* in 1995, 5 years by Etemadi *et al* in 1997, 8.62 years in a study by Etemadi *et al* in 2005 and 14.6 years by Etemadi *et al * in 2007 (11). According to the study by Etemadi in 1997, the course of the disease in 91.6% of the patients was chronic (more than 6 months), 3.88% subacute (11 days to 6 months) and 4.85% (less than 10 days) (11). According to a study by Etemadi in 2000, 92% of the patients had chronic and 8% had subacute progression. No acute progression was reported in this study (11). According to the study by Etemadi *et al* in 2005, 49.3% of the patients had a rapid advance early in the disease with steady symptoms afterwards (11). Boostani *et al* mentioned in 2010 that 87.5% of the patients had a clinical course of more than 2 years and the rest had a course of less than 2 years (11).

Neurological findings usually include symmetrical lower extremity weakness and spasticity (spastic paraparesis), sensory disturbances (especially in vibration and less commonly in proprioceptive senses) and sensory ataxia, impotence, bladder dysfunction (spastic bladder) and less frequently bowel dysfunction, and generalized hyperreflexia except in cases of concomitant sensory neuropathy (22-24). According to the study by Etemadi, *et al,*1994 and 1997, the most common complaints of HAM/TSP patients were lower limbs weakness, gait disorders, muscle weakness and urinary symptoms respectively (11). In other studies by Etemadi *et al* in 2000 and 2004, gait disorder and muscle weakness were the most common complaints (11). According to a study by Boostani *et al* in 2010 gait disorder and lower limbs’ weakness were the main symptoms, respectively (11). The most common signs in neurologic examination in all these studies were increased muscle tone in lower limbs and bilateral extensor response in planter reflex. Urinary complaints, constipation and sweating disorders were respectively the most common autonomic complaints in HAM/TSP patients according to the findings of Etemadi *et al* in 2005 (11). Moreover, according to the studies in 2004 and 2007, Etemadi *et al *mentioned that the most and the least common urinary complaints of the patients were frequency and incontinency, respectively. Furthermore, they demonstrated that 100% of HAM/TSP patients had increased diameter of urinary bladder tissue and also increased urinary residue. In urodynamic methods, 50% of the patients had decreased urinary bladder compliance, 70% involuntary contractions and detrusor instability, and 60% detrusor-sphincter dyssynergia. This study also showed that 34% of patients were not satisfied with their sex life and their sexual activity (11).

 Conditions that may clinically resemble HAM/TSP include HIV-associated vacuolar myelopathy, idiopathic inflammatory conditions and demyelinating diseases such as multiple sclerosis (especially the primary progressive type), hereditary spastic paraplegia (HSP), and toxic-metabolic disorders such as vitamin B12 and niacin deficiency, tropical ataxic neuropathy (TAN), adrenoleukodystrophy, primary lateral sclerosis (PLS), lathyrism in India, cycad poisoning in Pacific Islands, or konzo in Central parts of Africa (1, 7). According to the study conducted by Etemadi *et al *in 1997, HTLV-I infection prevalence in spastic paraparetic or spastic tetraparetic patients was 20.6% (11). This rate was 24.4% in another study by Etemadi *et al* in 2000 (11).

*Neurologic findings other than myelopathy*

Regarding the less common neurologic manifestations of HTLV-I infection, Etemadi *et al* reported one case of brainstem involvement, one case of polymyositis, one case of neuropathy, and one case of muscular atrophy in 2000 (11). Etemadi *et al* in 1994 reported 2 cases of pseudo ALS symptoms caused by HTLV-I infection (11). In a study by Etemadi *et al* in 1997, one case of polymyositis with HTLV-I infection and one case of facial paralysis were reported (11). In the same study, an ALS-like presentation was reported in 7.76% of the patients. Saeidi *et al* proved that 30.1% of HAM/TSP patients had degrees of peripheral nerve involvement with the most common form as axonal motor-sensory polyneuropathy using electrodiagnostic methods in 2005. This study also revealed that the patients who had breast-feeding for more than one year were less affected by neuropathy. In this study, the more was the movement disability of HAM/TSP patients, the more was the probability of peripheral neuronal involvement, 

*Non-neurological findings*

YazdanPanah *et al *in 2008 showed that there was a correlation between HTLV_-I _infection and recurrent oral aphtous ulcer, skin eczema and non-genital verruca (11). YazdanPanah *et al* in another study in 2009 showed a sixfold increase in the probability of developing skin lesions comparing to general population (11). According to this study, the probability of developing skin lesions is more likely in women infected with the virus than in men. It has been proved that patients with myelopathy caused by HTLV_-I _are more likely to develop skin lesions during the disease, especially in the second decade of the disease. Only skin coarsening had a significant correlation with HTLV-I in this study. The prevalence of other skin lesions did not have any meaningful difference in _HTLV-I _infected patients compared with the general population. Besides, adaptive skin response was apparently decreased in HAM/TSP patients compared with the control group. In 1997, Abrishami*, et al,* reported 19.2% HTLV-I infection among patients with idiopathic uveitis (11).

## Diagnosis

Diagnosis of HTLV-I infection is based on the detection of specific antibodies against the virus by enzyme-linked immunosorbent assay (ELISA) or agglutination tests (25, 26). Positive results should be confirmed by Western blot (WB) or Polymerase Chain Reaction (PCR) tests (27, 28). The establishment of the diagnosis of HAM/TSP requires HTLV-I-positive serology in blood and cerebrospinal fluid (CSF) (29, 30). If CSF reveals a combination of positive PCR test together with the evidence of an increased HTLV-I- specific antibody index and oligoclonal bands, it will be more sensitive and specific (20). According to a study by Etemadi *et al* in 1997, 25% of the patients had 50-100 mgr/dl protein in CSF and 70% had a normal protein (11). In 71% of the patients in this study, IgG was increased in CSF. According to a study accomplished by Etemadi *et al* in 2004, 66.3% of the patients showed pleocytosis in CSF, 44% of the patients had increased protein and 13.1% had decreased glucose (11).

 Imaging of the brain and spinal cord with MRI may be normal or may reveal spinal cord atrophy (especially in the thoracic cord) and hyperintense signal abnormalities on T2-weighted images in periventricular or subcortical white matter in the brain. The lesions may show some enhancement in post-contrast images, usually with a diffuse or irregular pattern, in contrast to discrete or multifocal enhancement seen in MS (1, 7, 19). A plaque around ventricle was reported in MRI of three HAM/TSP patients in a study by Etemadi *et al* in 2000 and another patient had a cervical plaque (11). In Etemadi’s study in 2004, 9.5% of the patients had abnormal MRI, and one case of cerebellar atrophy was reported in the MRI of a HAM/TSP patient (11). In a study by Boostani *et al* three infected patients had brain plaques, and all of them had less than three plaques in their imaging and all plaques were smaller than 1cm (11).

## Treatment

There is not any antiviral agents that can treat effectively HAM/TSP, but some immunomodulatory agents have some effects on the course of the disease (1, 31-33). Overall, treatment options are divided into symptomatic and etiologic groups. Symptomatic management includes antispastic and anticholinergic agents, analgesics, physiotherapy, and the management of emotional and social problems (34, 35). Etiological treatments include corticosteroids, cytotoxic agents, interferon alpha, plasma exchange (PE), and other immunomodulatory agents such as Danazol, Erythromycin, Phosphomycin, Sulphasalazine, and Pentoxyphillin. Early treatment, especially during the first year, provides the chance of improvement because of the more rapid progress of the disease during this time (36, 37).

 Various regimens for administrating corticosteroids have been experienced by different investigators, such as the initial IV pulse therapy with or without tapering, prolonged oral corticosteroid in a daily or every other day regimen, and even intrathecal corticosteroid. It has been effective in improving motor function to some extent. Patients who fail to respond to corticosteroid therapy or cannot tolerate it due to the side effects may have some improvements with PE or interferon alpha (7, 32, 38-40). Recently, interferon alpha has been suggested by some investigators as an appropriate substitute for corticosteroids due to fewer side effects (38). It has significant, but often temporary, improvement in motor and urinary abnormalities. The Anabolic Corticosteroid, Danazol, has been reported to decrease disability and improve some subjective symptoms such as gait difficulty, paresthesia and especially bladder symptoms (7). In 1997, Etemadi *et al* studied methylprednisolone pulse therapy on HAM/TSP patients and concluded that more than 90% of the patients had a relative to absolute satisfaction of the treatment (11). The most therapeutic effect in the first place was on the complaint of pain and in the second place was on the muscle weakness and gait disorder. Another study in 2004 by Etemadi *et al* showed that Danazol had a medium but statistically meaningful effect on the decrease of movement disability (11). The most therapeutic effect of Danazol was on spasticity and gait disorders and the least effects were on paraesthesiae and pain. In 2008, Azarpazhooh et-al. studied clinical and immunological effect of interferon alpha prescription on treatment of HAM/TSP patients (11). In this study, during a short course of 6-month treatment via interferon alpha, there was an improvement in force, urinary system function, spasticity and decrease in viral load, antibody level, lymphocytes and monocytes. Clinical improvement was associated with a change in immunological function to such a high degree that it confirmed the close relationship between virus pathogenesis and the immune system. Side effects of the interferon therapy were around 64% with fever and chills, weakness and malaise, alopecia and depression being the most common complications. In this study, one case of intracranial hemorrhage and one case of lupus like reaction were reported.
